# DNA methylation mediates the effect of maternal smoking during pregnancy on birthweight of the offspring

**DOI:** 10.1093/ije/dyv048

**Published:** 2015-04-10

**Authors:** Leanne K Küpers, Xiaojing Xu, Soesma A Jankipersadsing, Ahmad Vaez, Sacha la Bastide-van Gemert, Salome Scholtens, Ilja M Nolte, Rebecca C Richmond, Caroline L Relton, Janine F Felix, Liesbeth Duijts, Joyce B van Meurs, Henning Tiemeier, Vincent W Jaddoe, Xiaoling Wang, Eva Corpeleijn, Harold Snieder

**Affiliations:** ^1^Departments of Epidemiology,; ^2^Pulmonology and; ^3^Genetics, and; ^4^LifeLines Cohort Study, University Medical Center Groningen, Groningen, The Netherlands,; ^5^Georgia Regents University, Augusta, Georgia, USA,; ^6^MRC Integrative Epidemiology Unit, University of Bristol, Bristol, UK,; ^7^Departments of Epidemiology,; ^8^Pediatrics,; ^9^The Generation R Study Group and; ^10^Internal Medicine, Erasmus MC, University Medical Center Rotterdam, The Netherlands

**Keywords:** Epigenetic epidemiology, epigenome-wide association study, DOHaD, fetal programming, GECKO, ALSPAC, Generation R

## Abstract

**Background:** We examined whether the effect of maternal smoking during pregnancy on birthweight of the offspring was mediated by smoking-induced changes to DNA methylation in cord blood.

**Methods:** First, we used cord blood of 129 Dutch children exposed to maternal smoking vs 126 unexposed to maternal and paternal smoking (53% male) participating in the GECKO Drenthe birth cohort. DNA methylation was measured using the Illumina HumanMethylation450 Beadchip. We performed an epigenome-wide association study for the association between maternal smoking and methylation followed by a mediation analysis of the top signals [false-discovery rate (FDR) < 0.05]. We adjusted both analyses for maternal age, education, pre-pregnancy BMI, offspring’s sex, gestational age and white blood cell composition. Secondly, in 175 exposed and 1248 unexposed newborns from two independent birth cohorts, we replicated and meta-analysed results of eight cytosine-phosphate-guanine (CpG) sites in the *GFI1* gene, which showed the most robust mediation. Finally, we performed functional network and enrichment analysis.

**Results:** We found 35 differentially methylated CpGs (FDR < 0.05) in newborns exposed vs unexposed to smoking, of which 23 survived Bonferroni correction (*P* < 1 × 10^-7^). These 23 CpGs mapped to eight genes: *AHRR, GFI1, MYO1G, CYP1A1, NEUROG1, CNTNAP2, FRMD4A* and *LRP5*. We observed partial confirmation as three of the eight CpGs in *GFI1* replicated. These CpGs partly mediated the effect of maternal smoking on birthweight (Sobel *P* < 0.05) in meta-analysis of GECKO and the two replication cohorts. Differential methylation of these three *GFI1* CpGs explained 12–19% of the 202 g lower birthweight in smoking mothers. Functional enrichment analysis pointed towards activation of cell-mediated immunity.

**Conclusions:** Maternal smoking during pregnancy was associated with cord blood methylation differences. We observed a potentially mediating role of methylation in the association between maternal smoking during pregnancy and birthweight of the offspring. Functional network analysis suggested a role in activating the immune system.

**Key Messages**

- Maternal smoking during pregnancy is associated with genome-wide cord blood methylation differences.
- Differential methylation may mediate part of the association between maternal smoking during pregnancy and birthweight of the offspring.
- Functional network and enrichment analysis suggest a role in activating the immune system.
- Future research should include the collaboration of multiple birth cohorts to meta-analyse the (potentially mediating) role of differential methylation in early development.

## Introduction

It is well known that maternal smoking during pregnancy can cause intrauterine growth restriction and low birthweight.^1^^–^^4^ Low birthweight, in turn, has been associated with increased childhood growth and cardiometabolic problems in childhood and adulthood.^5^^,^^6^ The development of chronic diseases in adulthood is therefore believed to start during pregnancy as a result of exposure to adverse intrauterine environments, also known as fetal programming. We hypothesized that the long-lasting effects of adverse fetal exposures (e.g. smoking) on birthweight and subsequent cardiometabolic risk are at least partly caused by DNA methylation.^7^^–^^9^ Thus, maternal smoking during pregnancy may have adverse health consequences during the offspring’s entire life course via DNA methylation.

In recent studies, tobacco smoke exposure has been associated with DNA methylation changes in smokers.^10^^–^^12^ The effect of maternal tobacco smoking during pregnancy on DNA methylation of their offspring has also been investigated in a number of studies using different designs.^13^^–^^20^ Several of these studies in offspring investigated global or gene-specific DNA methylation differences, in umbilical cord blood and placental cells.^13^^,^^14^^,^^16^^,^^21^ Several studies have used an epigenome-wide association study (EWAS) design,^17^^–^^20^ focusing on methylation differences of individual cytosine-phosphate-guanine (CpG) sites. Some EWASs used the 27 k chip (Illumina Inc., San Diego, USA) in placental samples or whole blood samples of children and identified methylation of several CpGs to be associated with maternal smoking during pregnancy.^19^^,^^20^ Other EWASs used the 450 K chip (Illumina Inc., San Diego, USA) to identify changes in methylation associated with maternal smoking during pregnancy.^17^^,^^18^ Joubert *et al*.^17^ identified and replicated methylation changes in cord blood of several genes (*AHRR*, *CYP1A1* and *GFI1*) associated with maternal smoking during pregnancy. More recently, Markunas *et al*.^18^ identified and replicated differential methylation of CpGs in 10 novel genes in whole blood from 889 newborns. Other EWASs studied associations with birthweight.^22^^,^^23^ Adkins *et al*.^23^ found no epigenome-wide associations with birthweight, whereas Engel *et al*.^22^ identified 19 CpGs. Interestingly, no studies investigated mediation by methylation in the association between maternal smoking and birthweight or other health-related outcomes.

Therefore, we conducted an EWAS in cord blood to examine the association between maternal smoking during pregnancy and DNA methylation, with the 450 K chip. Furthermore, we studied for the first time whether differentially methylated CpGs mediated the effect of smoking on birthweight. Finally, we sought to replicate the most promising mediation findings in two independent birth cohorts, and meta-analysed the results.

## Methods

### Subjects

We derived data from GECKO Drenthe, a Dutch population-based birth cohort that studies risk factors associated with the development of overweight from birth into adulthood.^24^ The cohort includes 2874 children born between April 2006 and April 2007. Children have been extensively phenotyped on parental characteristics, pregnancy and delivery, children’s health, nutrition and childhood growth. Data were gathered during pregnancy and at multiple time points during childhood. Maternal and paternal smoking during pregnancy were self-reported and (if available) additional information from obstetricians was used. Directly after delivery, umbilical cord blood was collected from 1565 children and stored at -80°C. DNA was extracted from the buffy coats using the QIAamp96 DNA Blood Kit (QIAGEN). To increase DNA concentration to ≥ 50 ng/µl, all samples were treated with Glycoblue.

From all children in the total cohort with stored cord blood, we selected those that had sufficient DNA of good quality after DNA isolation (DNA concentration ≥ 50 µg/ml). Of those, we excluded non-Dutch newborns, premature newborns (≤37 weeks), twins and those with a mother with (gestational) diabetes. We also excluded children with missing information on these variables, which resulted in *n* = 1118. Then 447 children were selected because they had information on maternal and paternal smoking during pregnancy and the number of cigarettes smoked by the mother. This resulted in 129 children exposed to maternal smoking and 318 children unexposed to either maternal or paternal smoking. This group of 447 did not differ from the group of 1118 on gestational age, birthweight, maternal educational level or gender. Only the maternal pre-pregnancy BMI of the group of 447 was slightly lower (24.4 vs 25.0 kg/m^2^). Therefore, we concluded that these 447 were broadly representative of the total cohort. We used the complete exposed group (*n* = 129) and randomly selected 129 unexposed newborns (of which 3 dropped out during QC), see flowchart in Supplementary Figure S1, available as Supplementary data at *IJE* online.

This study has been approved by the Medical Ethics Committee of the University Medical Center Groningen, and parents of all participants gave written informed consent.

### Genome-wide methylation assay

We used 500 ng DNA per sample to perform methylation analysis. To minimize batch effects, we randomized all samples on sex and exposure status per chip over three 96-well plates. Thus each chip contained three exposed boys, three unexposed boys, three exposed girls and three unexposed girls. In addition, we randomly assigned five control samples of the same male to each plate; two on the first plate, two on the second plate and one on the third plate. We performed bisulphite conversion using the EZ-96 DNA methylation kit (Zymo research Corporation, Irvine, USA). After validating that unmethylated cytosines had converted to thymidines using commercially available bisulphite conversion controls (Zymo Research Corporation, Irvine, USA), we processed the samples using the Infinium HumanMethylation450 BeadChip (Illumina Inc., San Diego, USA). We checked performance of built-in internal quality controls in the Controls Dashboard in the methylation module of GenomeStudio (Illumina Inc., San Diego, USA).

### Quality control

For all 485 577 CpGs we calculated beta-values and detection *P*-values using the Minfi R package.^25^ Overall, beta-values ranged from zero to one, showing the level of methylation for each CpG, and detection *P*-values < 0.05 indicated that the target sequence signal was distinguishable from the background. We performed all quality control steps for the three plates separately. Cluster plots for the betas on the X chromosome showed a clear distinction by sex. Two males were in the female cluster, and were excluded from further analyses. Illumina-suggested background normalization and colour correction were performed. One sample did not meet the criterion of ≥ 99% of the CpGs with detection *P*-value < 0.05 and was excluded. This resulted in a final sample of 255 children: 129 exposed and 126 unexposed. Control probes, probes on X or Y chromosomes and probes that did not meet our criteria of a detection *P*-value of < 0.05 in ≥ 99% of the samples were excluded. This resulted in 465 891 remaining CpGs. The five duplicate male control samples (included in each plate) showed high correlations ranging from 0.995 to 0.998, indicating that batch effects were minimal. These five samples were removed from further analyses.

### Statistical analyses

We performed all pre-processing steps using R packages SWAN (Subset-quantile Within Array Normalization) and Minfi^25^ and linear regression in the R package Limma (Linear Models for Microarray Analysis).^26^ We generated basic characteristics, mediation analysis and the volcano plot in Stata v12 (StataCorp, College Station, TX, USA).

### Epigenome-wide association (EWAS) analysis

We performed linear regression analyses in Limma comparing the methylation beta values of the exposed with the unexposed group. We adjusted for the following covariates that were selected based on their expected association with maternal smoking and/or methylation: sex, gestational age, maternal age, pre-pregnancy BMI, educational level, plate number and cell type composition.^17^^,^^18^^,^^27^ Sex and gestational age (weeks) were reported by obstetricians. Maternal educational level (low/average vs university educated), maternal BMI before pregnancy (kg/m^2^) and maternal age (years) were self-reported by the mothers. Missing values on gestational age (*n* = 2), maternal educational level (*n* = 3) and maternal pre-pregnancy BMI (*n* = 8) were imputed with the mean/median to maintain power. Excluding the 10 newborns with ≥ 1 missing covariate did not alter the results, and since multiple imputation in an EWAS dataset would be computationally burdensome, we present our findings including these 10 samples with single imputed covariate data. Furthermore, the number of participants with missing data was very small, thus substantial bias was unlikely. Additionally, we included plate number to adjust for potential batch effects and we calculated cell type proportions based on the method previously presented by Houseman and colleagues^28^ with the dataset presented by Reinius and colleagues.^29^ These cell type proportions (B cells, granulocytes, monocytes, NK cells, CD4+ T cells and CD8+ T cells) were included as covariates in the model. As a sensitivity analysis, we also performed our analysis without correction for cell type and even in a crude model without any of the covariates, to test the effect of these covariates on our results. We converted raw *P*-values to false discovery rates (FDRs) based on Benjamini and Hochberg.^30^ We used both FDR < 0.05 (raw *P* < 7.5 × 10^-6^) and Bonferroni corrected *P*-values (raw *P* < 1 × 10^-7^) as significance thresholds. We tested a dose-response effect of number of cigarettes per day on methylation in the exposed group for those signals with FDR < 0.05.

### Mediation analysis

**Figure 1.**
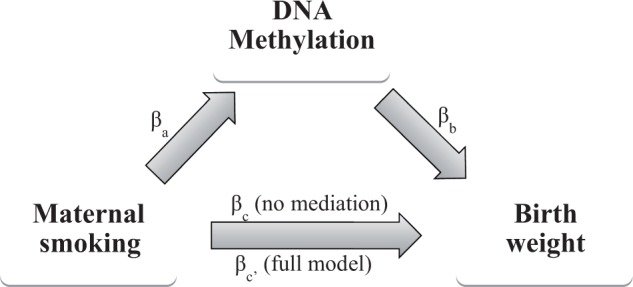
Hypothetical mediation model explaining the variables in the mediation analysis. β_a_, effect estimate for smoking in the model: CpG = smoking + covariates. β_b_, effect estimate for CpG in the model: BW = CpG + covariates. β_c_, effect estimate for smoking in the model: BW = smoking + covariates. β_c’_: effect estimate for smoking in the model: BW = smoking + CpG + covariates.

We tested the CpGs with FDR < 0.05 for mediation in the association between maternal smoking during pregnancy and birthweight, using the widely used method of Baron and Kenny^31^ and the Sobel test.^32^ As shown in Figure 1, mediation was considered to be present when: (i) smoking correlated with methylation level (*βa*); (ii) smoking correlated with birthweight without adjusting the model for the mediator (*βc*); (iii) differential methylation correlated with birthweight (*βb*); (iv) the association between smoking and birthweight decreased upon addition of methylation to the model (*βc’*); and (v) the Sobel test gave *P* < 0.05, indicating a decrease in the effect of smoking on birthweight after adjusting for the differentially methylated CpG. For those CpGs showing mediation, we tested the assumption that there is no interaction of the exposure and covariates with the mediator CpGs.^33^^,^^34^ For the mediating CpGs, we further calculated which part of the association between smoking and birthweight could be explained by the mediator using the formula:^35^

$$\left(\beta_{\text{a}}{\text{*β}}_{\text{b}}\right)\text{/}\left(\left(\beta_{\text{a}}{\text{*β}}_{\text{b}}\right){\text{+β}}_{\text{c′}}\right)\text{.}$$

The mediation effect β_a_*β_b_ equals β_c_ - β_c’_, thus this formula equals:

$$\beta_{\text{c}}{\text{-β}}_{\text{c′}}{\text{/β}}_{\text{c}}\text{.}$$

### Functional network analysis

We performed network and enrichment analysis to facilitate the functional interpretation of our differentially methylated genes using GeneMANIA.^36^^,^^37^ To this end, we selected all genes to which the top CpGs (FDR < 0.05) mapped as input, to construct a functional interaction network by adding the 100 most strongly interacting genes. Data resources used by the GeneMANIA algorithm were functional association datasets including genetic interactions, protein-protein, co-expression, shared protein domains and co-localization networks.^36^^,^^38^ Functional enrichment analysis of all genes of the constructed interaction network against Gene Ontology (GO) terms was performed to find the most enriched GO terms.

### Replication

We performed replication analyses for the top findings of our EWAS and mediation analysis in two independent birth cohorts with 450 K methylation data in cord blood samples from Caucasian children: ALSPAC (Avon, UK)^39^^,^^40^ and Generation R (Rotterdam, The Netherlands^41^). For the replication analyses, we analysed data of 65 exposed and 613 unexposed offspring in ALSPAC and 110 exposed and 635 unexposed offspring in Generation R (see Supplementary text and Supplementary Table S1, available as Supplementary data at *IJE* online). All eight *GFI1* CpGs with FDR < 0.05 in the EWAS were taken forward for replication. We limited replication to the *GFI1* gene as its CpGs showed the most robust and clearest mediation results and *GFI1* was among the genes with the most robust EWAS signals in GECKO. Furthermore, unlike *NEUROG1*, differential methylation of *GFI1* was previously reported to be associated with maternal smoking.^17^ Exposure in the replication cohorts was defined as sustained maternal smoking during pregnancy vs no maternal smoking during pregnancy, because this was the most accurate measure of exposure in the replication cohorts. Paternal smoking was adjusted for in the mediation analysis. Except for this additional covariate, mediation analyses were performed using the same analysis protocol as in GECKO. In order to obtain one overall estimate of the results for each of the eight *GFI1* CpGs, we used fixed effects inverse variance meta-analysis of the results of the two replication cohorts. Subsequently, we combined results of discovery (GECKO) and replication (ALSPAC and Generation R) stages in a joint meta-analysis. We concluded that mediation was present for CpGs showing a two-sided *P* < 0.05 in both the replication and the joint meta-analysis.

## Results

General characteristics of all participants in GECKO are presented in Table 1, for characteristics of ALSPAC and Generation R participants see Supplementary Table S1, available as Supplementary data at *IJE* online. On average, in GECKO, smoking mothers were 1.4 years younger and more often had a lower educational level and their children had a 281 g lower birthweight.

**Table 1.** Characteristics of children exposed and unexposed to maternal smoking (*n* = 255 in GECKO)

Characteristics	Unexposed (*n* = 126)	Exposed (*n* = 129)	*P*_difference_
Male	66 (52.4)	70 (54.3)	0.76
Birthweight	3685 ± 563	3404 ± 464	<0.0001
Gestational age	39.8 ± 1.2	39.7 ± 1.3	0.18
Maternal age at childbirth	31.1 ± 3.6	29.7 ± 4.7	<0.01
Maternal low/middle educational level	70 (55.6)	105 (81.4)	<0.0001
Maternal pre-pregnancy BMI	23.9 ± 3.3	24.9 ± 5.1	0.09
Number of cigarettes smoked	NA	10 (1–30)	

Data shown as *n* (%) or mean ± SD. Except for number of cigarettes smoked: median (range). *P-*values are given for independent samples t-test (continuous) or chi-square test (categorical).

Unexposed group was defined as no smoking during pregnancy, by mother or by father. Exposed group was defined as smoking during pregnancy by mother.

We found 35 CpGs, mapping to 10 genes, that showed differential methylation (FDR < 0.05) between the groups exposed and unexposed to maternal smoking (Table 2). After the more conservative Bonferroni correction, 23 CpGs remained. These 23 CpGs mapped to eight genes: *AHRR*, *GFI1*, *MYO1G*, *CYP1A1*, *NEUROG1*, *CNTNAP2*, *FRMD4A* and *LRP5*. All eight CpGs mapping to *GFI1*, *LRP5* and *CNTNAP2* had lower methylation levels in the group exposed to maternal smoking during pregnancy compared with the unexposed group (methylation difference (beta value exposed minus beta value unexposed) ranged from −0.021 to −0.117). The 11 CpGs that mapped to *MYO1G*, *NEUROG1, FRMD4A* and *CYP1A1* had higher methylation levels in the exposed group (methylation difference ranged from 0.028 to 0.077). For *AHRR*, three CpGs had lower methylation levels (methylation difference between −0.024 and −0.073) whereas one had higher methylation in the exposed group (methylation difference 0.038).

**Table 2.** Top 35 CpGs with methylation difference between children exposed and unexposed to maternal smoking (FDR < 0.05)

CpG	Closest gene	Chr	Bp position	Location in gene	Located in island, shore or open sea	Mean methylation percentage	Methylation difference	*P*-value
cg05575921	*AHRR*	5	373378	Body	Shore	0.688	−0.073	1.14E-25
cg04180046	*MYO1G*	7	45002736	Body	Island	0.497	0.056	1.10E-14
cg09935388	*GFI1*	1	92947588	Body	Island	0.661	−0.105	2.67E-14
cg11429111	*NEUROG1*^a^	5	134813329	−	Open sea	0.690	0.048	7.17E-12
cg14179389	*GFI1*	1	92947961	Body	Island	0.188	−0.061	1.76E-11
cg12803068	*MYO1G*	7	45002919	Body	Shore	0.759	0.077	1.79E-11
cg12876356	*GFI1*	1	92946825	Body	Island	0.660	−0.107	1.79E-11
cg01952185	*NEUROG1*^a^	5	134813213	–	Open sea	0.590	0.047	3.32E-11
cg18146737	*GFI1*	1	92946700	Body	Island	0.739	−0.117	3.81E-11
cg22132788	*MYO1G*	7	45002486	Body	Island	0.881	0.053	1.57E-10
cg23067299	*AHRR*	5	323907	Body	Shore	0.723	0.038	2.66E-10
cg21611682	*LRP5*	11	68138269	Body	Open sea	0.519	−0.021	2.83E-10
cg18316974	*GFI1*	1	92947035	Body	Island	0.784	−0.102	3.27E-10
cg15507334	*FRMD4A*	10	14372913	TSS200	Open sea	0.556	0.028	2.90E-09
cg05549655	*CYP1A1*	15	75019143	TSS1500	Island	0.256	0.036	3.20E-09
cg19089201	*MYO1G*	7	45002287	3'UTR	Island	0.796	0.037	3.53E-09
cg11924019	*CYP1A1*	15	75019283	TSS1500	Island	0.473	0.036	9.04E-09
cg09662411	*GFI1*	1	92946132	Body	Island	0.714	−0.066	9.55E-09
cg25949550	*CNTNAP2*	7	145814306	Body	Shore	0.136	−0.022	9.84E-09
cg22549041	*CYP1A1*	15	75019251	TSS1500	Island	0.304	0.052	1.53E-08
cg14817490	*AHRR*	5	392920	Body	Open sea	0.336	−0.030	3.98E-08
cg21161138	*AHRR*	5	399360	Body	Open sea	0.742	−0.024	6.18E-08
cg18092474	*CYP1A1*	15	75019302	TSS1500	Island	0.564	0.047	9.24E-08
cg22937882	*AHRR*	5	405774	Body	Open sea	0.857	0.016	2.23E-07
cg25464840	*FRMD4A*	10	14372910	TSS200	Open sea	0.675	0.025	3.07E-07
cg24159436	*PLCL2*	3	16974681	1stExon	Open sea	0.622	0.028	1.10E-06
cg04535902	*GFI1*	1	92947332	Body	Island	0.797	−0.057	1.77E-06
cg12101586	*CYP1A1*	15	75019203	TSS1500	Island	0.383	0.040	2.11E-06
cg11813497	*FRMD4A*	10	14372879	TSS200	Open sea	0.700	0.028	4.01E-06
cg01970407	*AHRR*	5	323320	Body	Shore	0.678	0.023	4.07E-06
cg13834112	*–*	15	90361639	–	Shelf	0.625	0.028	4.54E-06
cg23680900	*CYP1A1*	15	75017924	TSS200	Shore	0.149	0.015	5.01E-06
cg17292337	*–*	12	31272112	–	Open sea	0.361	−0.098	5.14E-06
cg01264106	*LGALS1*	22	38071602	TSS200	Shore	0.346	0.020	5.78E-06
cg10399789	*GFI1*	1	92945668	Body	Shore	0.738	−0.049	7.48E-06

Analyses were corrected for plate, sex, gestational age, maternal age, maternal education, maternal BMI and cell type composition.

Methylation difference was calculated from the average beta values of exposed minus unexposed groups.

^a^Closest gene was NEUROG1 (57 411–57 527 bp downstream), all other CpGs were mapped within the boundaries of the given genes.

Effects of covariate adjustment on EWAS results are shown in Supplementary Table S2, available as Supplementary data at *IJE* online. Analysis without adjustment for cell type distribution did not substantially change our top (Bonferroni significant) findings, but the list of CpGs with FDR < 0.05 decreased substantially after cell type correction.

**Figure 2.**
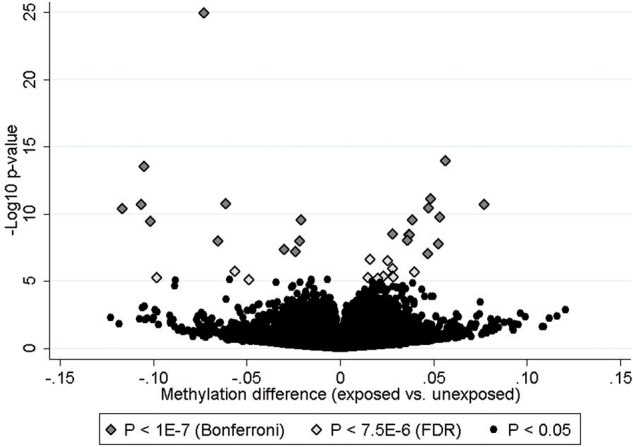
Volcano plot showing methylation differences between exposed and unexposed against –log_10_ of the *P*-values.

The volcano plot in Figure 2 shows the methylation differences between the exposed and unexposed groups plotted against statistical significance. It shows the 35 differentially methylated CpGs (FDR < 0.05) and the 23 CpGs that remained statistically significant after Bonferroni correction.

We observed no dose-response effect of number of cigarettes smoked per day on differential methylation in the exposed group for any of the 35 top CpGs (data not shown).

Next, we considered the 35 top CpGs to test for the mediating effect in the association between maternal smoking and birthweight. All CpGs on the growth factor independent 1 transcription repressor (*GFI1*) gene (eight CpGs) and the neurogenin 1 (*NEUROG1*) gene (two CpGs) showed mediation with *P* < 0.07 in GECKO (Table 3), whereas the other CpGs did not (Supplementary Table S3, available as Supplementary data at *IJE* online). None of these CpGs showed interaction with the exposure or covariates in its effect on birthweight (Supplementary Tables S4a–h, available as Supplementary data at *IJE* online). We limited replication analysis to CpGs in *GFI1* because it showed the most robust results.

**Table 3.** Mediation analysis examining the indirect effect of maternal smoking during pregnancy on birthweight through methylation in GECKO

	**β_c_**	**SE_c_**	**P_c_**	**R-square**			

**BW = smoking + covariates**	−264.3	59.4	1.3E-05	0.307			

**BW = smoking + CpG + covariates**	**β_c’_**	**β_b_**	**SE_b_**	**P_b_**	**Difference in betas (β_c_ - β_c’_)**	**Mediation percentage ((β_c_ - β_c’_) / β_c_)**	**Sobel *P*-value**
*GFI1*							
cg09935388	−143.3	1190.4	294.3	7.0E-05	−121.0 g	45.8%	0.0003
cg14179389	−214.4	820.7	427.0	5.6E-02	−49.9 g	18.9%	0.064
cg12876356	−158.0	970.4	253.4	1.6E-04	−106.3 g	40.2%	0.001
cg18146737	−165.6	856.3	227.7	2.1E-04	−98.7 g	37.3%	0.001
cg18316974	−177.7	841.3	248.7	8.0E-04	−86.6 g	32.8%	0.002
cg09662411	−196.5	1025.5	346.9	3.4E-03	−67.8 g	25.7%	0.008
cg04535902	−193.3	1222.9	324.1	2.0E-04	−71.0 g	26.9%	0.002
cg10399789	−217.3	1023.2	355.3	4.3E-03	−47.0 g	17.8%	0.018
*NEUROG1*							
cg11429111	−202.2	−1436.3	580.3	0.014	−62.1 g	23.5%	0.019
cg01952185	−219.4	−1161.2	560.5	0.039	−44.9 g	17.0%	0.052

BW, birthweight.

Covariates: plate, sex, gestational age, maternal age, maternal education, maternal BMI and cell type composition.

Sobel test = β_c_ − β_c’_ / SE, where SE = $\surd {\text{(β}}_{\text{a}}{}^{\text{2}}{\text{*SE}}_{\text{b}}{}^{\text{2}}{\text{+β}}_{\text{b}}{\text{2*SE}}_{\text{a}}{}^{\text{2}}\text{)}$.

The coefficients βc and βc’ can be interpreted as the amount of grams lower birthweight for smoking vs non-smoking mothers in the ‘smoking to birthweight’ and full model, respectively. βb represents the effect of methylation level (coded as a proportion between 0–1) on birthweight. For cg09935388 this means that an increase of 100% in methylation level is associated with 1190.4 g higher birthweight. For extra information on the betas, see Figure 1.

Replication and meta-analysis in ALSPAC and Generation R confirmed the association with maternal smoking for seven of the eight CpGs in *GFI1* and mediation was replicated for three of the eight *GFI1* CpGs: cg09935388, cg14179389 and cg12876356 (Table 4). Although not all these CpGs were significant in the two individual replication cohorts, directions of the effects were consistent (Supplementary Table S5, available as Supplementary data at *IJE* online). Joint meta-analysis of discovery and replication cohorts combined showed that differential methylation of these three *GFI1* CpGs explained 12–19% of the 202 g lower birthweight in smoking mothers. For example, this was 19% for cg09935388 calculated as follows: newborns of smoking mothers had a 202 g lower birthweight compared with unexposed newborns (meta-analysis of β_c_, data not shown). After adding the CpG as mediator in the model, the effect of smoking on birthweight decreased by 37.5 g (β_c_ − β_c’_ in overall meta-analysis, see Table 4). Therefore, 37.5/202 = 19% of the 202 g lower birthweight in exposed newborns could be explained by mediation through differential methylation.

**Table 4.** Results of meta-analysis (EWAS and mediation model) for GECKO, ALSPAC and Generation R

***Epigenome-wide association study***						
	***Discovery***		***Replication meta-analysis***		***Overall meta-analysis (disc. + repl.)***	
	GECKO		ALSPAC & Generation R		GECKO, ALSPAC & Generation R	
CpG	Methylation difference	*P*-value	Methylation difference	*P*-value	Methylation difference	*P*-value
cg09935388	−0.105	2.67E-14	−0.103	2.30E-19	−0.104	4.61E-32
cg14179389	−0.061	1.76E-11	−0.064	2.54E-17	−0.063	3.13E-27
cg12876356	−0.107	1.79E-11	−0.086	1.20E-14	−0.093	2.48E-24
cg18146737	−0.117	3.81E-11	−0.098	1.24E-14	−0.105	4.53E-24
cg18316974	−0.102	3.27E-10	−0.060	2.35E-07	−0.074	4.00E-15
cg09662411	−0.066	9.55E-09	−0.049	8.52E-09	−0.055	8.88E-16
cg04535902	−0.057	1.77E-06	−0.00925	3.15E-01	−0.027	2.04E-04
cg10399789	−0.049	7.48E-06	−0.030	3.04E-03	−0.039	1.75E-07

***Mediation analysis***									
	***Discovery***			***Replication meta-analysis***			***Overall meta-analysis (disc. + repl.)***		
	GECKO			ALSPAC & Generation R			GECKO, ALSPAC & Generation R		
CpG	Δ beta (β_c_ - β_c’_)	Mediation % ((β_c_ - β_c’_) / β_c_)	Sobel *P*-value	Δ beta (β_c_ - β_c’_)	Mediation % ((β_c_ - β_c’_) / β_c_)	Sobel *P*-value	Δ beta (β_c_ - β_c’_)	Mediation % ((β_c_ - β_c’_) / β_c_)	Sobel *P*-value
**cg09935388**	**−121.0 g**	**45.8%**	**0.0003**	**−28.7 g**	**16.2%**	**0.0081**	**−37.5 g**	**18.6%**	**0.0003**
**cg14179389**	**−49.9 g**	**18.9%**	**0.064**	**−21.3 g**	**12.0%**	**0.0436**	**−25.1 g**	**12.4%**	**0.0107**
**cg12876356**	**−106.3 g**	**40.2%**	**0.001**	**−29.9 g**	**16.8%**	**0.0061**	**−38.1 g**	**18.9%**	**0.0002**
cg18146737	−98.7 g	37.3%	0.001	−19.6 g	11.0%	0.1143	−31.3 g	15.5%	0.0062
cg18316974	−86.6 g	32.8%	0.002	3.4 g	−1.9%	0.7032	−4.7 g	2.3%	0.5844
cg09662411	−67.8 g	25.7%	0.008	−8.5 g	4.8%	0.3730	−15.7 g	7.8%	0.0788
cg04535902	−71.0 g	26.9%	0.002	1.5 g	−0.8%	0.8107	−3.5 g	1.7%	0.5689
cg10399789	−47.0 g	17.8%	0.018	−4.5 g	2.5%	0.5237	−9.4 g	4.7%	0.1611


Disc, discovery; repl, replication.

For all meta-analysis we have used a two-sided *P* < 0.05 as significance threshold.

Bold: CpG sites for which significant mediation was confirmed (*P* < 0.05 for both replication meta-analysis and overall meta-analysis).

We observed 28 enriched GO terms (FDR < 0.05) for the 110 genes in the interaction network (Table 5). Most enriched terms are closely related and point towards regulation of immune system processes, particularly the cell-mediated immunity response.

**Table 5.** Enriched gene ontology terms identified in functional network analysis

GO ID	Description	FDR	Occurrences in sample	Occurrences in genome
GO:0046649	Lymphocyte activation	1.87 e-07	16	294
GO:0042110	T cell activation	2.11E-07	14	217
GO:0042101	T cell receptor complex	3.07E-07	6	13
GO:0050900	Leukocyte migration	1.21e-06	13	214
GO:0050851	Antigen receptor-mediated signalling pathway	2.09e-06	10	108
GO:0002429	Immune response-activating cell surface receptor signalling pathway	2.98e-06	10	114
GO:0050852	T cell receptor signalling pathway	3.77E-06	9	86
GO:0002768	Immune response-regulating cell surface receptor signalling pathway	4.73e-06	10	123
GO:0002757	Immune response-activating signal transduction	9.00e-05	11	219
GO:0043235	Receptor complex	9.00e-05	9	128
GO:0002764	Immune response-regulating signalling pathway	1.29e-04	11	229
GO:0002253	Activation of immune response	4.75e-04	11	263
GO:0030098	Lymphocyte differentiation	5.31e-04	8	119
GO:0002696	Positive regulation of leukocyte activation	5.31e-04	9	164
GO:0030217	T cell differentiation	5.31E-04	7	82
GO:0050867	Positive regulation of cell activation	6.38e-04	9	170
GO:0002521	Leukocyte differentiation	1.66e-03	9	192
GO:0051249	Regulation of lymphocyte activation	2.02e-03	9	198
GO:0051251	Positive regulation of lymphocyte activation	2.60e-03	8	153
GO:0002274	Myeloid leukocyte activation	2.71e-03	6	70
GO:0002694	Regulation of leukocyte activation	4.27e-03	9	221
GO:0050865	Regulation of cell activation	7.94e-03	9	240
GO:0043230	Extracellular organelle	1.90e-02	5	61
GO:0070062	Extracellular vesicular exosome	1.90e-02	5	60
GO:0065010	Extracellular membrane-bounded organelle	1.90e-02	5	61
GO:0002250	Adaptive immune response	1.95e-02	6	103
GO:0001773	Myeloid dendritic cell activation	2.63e-02	3	12
GO:0050863	Regulation of T cell activation	2.63E-02	7	162

GO ID, gene ontology identification number.

## Discussion

We aimed to examine the effect of maternal tobacco smoking during pregnancy on DNA methylation in cord blood. Our second aim was to study the mediating effect of DNA methylation in the association between maternal smoking during pregnancy and offspring’s birthweight. We found 35 CpGs (FDR < 0.05) in 10 genes to be differentially methylated in the exposed and non-exposed groups; 23 of these CpGs (in eight genes) survived Bonferroni correction. Furthermore, replication analysis confirmed methylation of three *GFI1* CpGs to mediate the association between maternal smoking during pregnancy and decreased birthweight. Finally, functional network analysis showed that the top differentially methylated genes influenced immune system processes, particularly related to cell-mediated immunity.

The association between smoking and methylation is one of the most widely studied epigenetic associations and evidence from EWASs on maternal tobacco smoking and DNA methylation specifically in offspring is accumulating rapidly.^13^^–^^20^ EWASs investigating the influence of cigarette smoking have used a variety of DNA sources, including placental cells,^19^ and studies in active smokers have been performed in whole blood, peripheral blood, lymphoblast DNA or lung alveolar macrophages^10^^–^^12^ with a generally high level of consistency across tissue and studies. To our knowledge only a limited number of EWASs have been published investigating the effect of maternal smoking during pregnancy in offspring using the 450 K chip, of which only one was done in cord blood.^17^^,^^18^

The 23 differentially methylated CpGs mapped to eight genes: *AHRR*, *GFI1*, *MYO1G*, *CYP1A1*, *NEUROG1*, *CNTNAP2*, *FRMD4A* and *LRP5*. Differential methylation of these genes (except for *NEUROG1*) was also observed (but not all consistently replicated) in other EWASs in cord and whole blood^17^^,^^18^ and/or in other studies into smoking and methylation in adults.^10^^,^^12^^,^^15^ Previous studies related methylation in the aryl-hydrocarbon receptor repressor (*AHRR*) gene and the Cytochrome P450, family 1, subfamily A1 (*CYP1A1*) gene to tobacco smoke exposure in both smokers and newborns and most studies, including ours, reported the same CpG as the top signal (cg05575921).^10^^,^^12^^,^^15^^,^^42^^,^^43^ Both *AHRR* and *CYP1A1* are involved in the aryl-hydrocarbon receptor (AhR) pathway, regulating the biological responses to hydrocarbons found in cigarette smoke and xenobiotic metabolism in general.^43^^–^^45^ The myosin-1 G (*MYO1G*) gene is involved in haematopoietic processes and regulation of cell elasticity.^46^ The contactin-associated protein-like 2 (*CNTNAP2*) gene is involved in the development of the nervous system^47^ and in neuropsychiatric disorders. Finally, the low-density lipoprotein receptor-related protein 5 (*LRP5*) gene plays a role in skeletal homeostasis.^48^ Differential methylation of the FERM Domain Containing 4A (*FRMD4A*) gene has also previously been observed in relation to tobacco smoke exposure in offspring of smoking mothers (in whole blood).^18^ Interestingly, single nucleotide polymorphisms in *FRMD4A* have been shown to be involved in nicotine dependence.^49^

An important finding in our study was the mediating effect of differential methylation of the growth factor independent 1 transcription repressor (*GFI1*) gene in the association between maternal smoking and birthweight. *GFI1* is known to play a role in developmental processes such as haematopoiesis and oncogenesis.^50^^,^^51^ Thus, *GFI1* could be involved in cellular development and possibly fetal growth. However, it has not previously been linked to birthweight or other anthropometric measures.

Differential methylation of *NEUROG1* also seemed to mediate the association between maternal smoking and birthweight in GECKO; however, our discovery results in *NEUROG1* await future replication. *NEUROG1* is known to be associated with neuronal differentiation and neurogenesis,^52^ making a link to fetal development plausible. It should be noted that these CpGs were not mapped within the *NEUROG1* gene regions, but located close to this gene (57 k downstream).

To the best of our knowledge, we were the first to investigate and identify statistical evidence of mediation by DNA methylation (in *GFI1*) in the pathway from maternal tobacco smoking during pregnancy to decreased birthweight of the offspring. Meta-analysis of all three cohorts showed that three CpGs on *GFI1* explained between 12% and 19% of the effect of maternal smoking on birthweight. These findings are promising, as this biological mechanism seemed to explain part of the effect of smoking on birthweight. Other mechanisms causing reduced fetal growth may involve impaired placental perfusion, chronically low levels of fetal oxygen supply^53^ and sensitivity to adipocytokines, e.g. leptin or ghrelin.^54^ However, it should be kept in mind that many other factors are involved in intrauterine growth and birthweight, e.g. malnutrition or stress,^55^^,^^56^ and that DNA methylation could not explain the total variation in birthweight resulting from smoking. As in any epidemiological study, residual confounding could not be entirely excluded. However, maternal smoking during pregnancy is known to have a direct adverse effect on growth of the fetus and is therefore likely to have a much stronger effect on methylation than other possible confounding factors.

We performed network and enrichment analysis to facilitate the functional interpretation of our 10 differentially methylated genes. Most enriched GO terms were related to immune system processes, especially to those related to cell-mediated immunity. Thus, intrauterine exposure to components in cigarette smoke seemed to elicit an immune response in the offspring. Such an immune response in smokers and offspring of smoking mothers may play a role in the increased risk of developing asthma.^57^ This is in line with studies showing that the AhR pathway activates the immune system triggered by environmental exposures such as tobacco smoke, pollutants and diet.^58^^,^^59^ Additional research will be needed to show whether these smoking-induced methylation effects may increase the risk of developing autoimmune diseases.^60^^–^^62^ These results seemed independent of cell type differences caused by maternal smoking, as we have adjusted all our analyses for these differences, although we cannot entirely exclude that cell correction was incomplete and residual cell (sub)type effects could be possible.

The current study has many strengths. We found that 78% of our top CpG signals overlapped with those from a previous EWAS on the same topic (data not shown), which is a testament to the robustness of our findings.^17^ Moreover, cord blood is an excellent tissue to test for methylation differences associated with maternal smoking, because cord blood has not yet been exposed to external influences other than those provided by the intrauterine environment. As such, potential confounding by those external exposures on the newborn is minimized. Use of cord blood to study DNA methylation as a potential mediator of birthweight is less ideal, as it implicitly assumes that it reflects methylation patterns from other tissues such as muscle, fat and bone that might be more plausibly causally related to fetal growth and birthweight. However, such tissues would be prohibitively difficult to collect from newborns and for this reason cord blood is currently the most commonly used tissue in epidemiological studies of newborns.^63^ Furthermore, in (epi)genetic epidemiology the winner’s curse is a well-known phenomenon, which means that the effect sizes of newly identified associations are often overestimated in the discovery cohort. For this reason we reported effect sizes of the combined analyses of discovery and replication cohorts, which showed only partial replication of our discovery findings. We were able to replicate three of the eight mediating CpGs in two other cohorts, which confirmed and strengthened our results. However it should be kept in mind that not all CpGs replicated and those CpGs that did replicate did not show such strong mediation as in the discovery sample. Another strength was the inclusion of the mediation analysis, giving more insight into the biological pathway between maternal smoking and birthweight.

To our knowledge, this is the first study to formally assess and report this mediating effect of DNA methylation. Additionally, we gave a functional interpretation of our results using functional network and enrichment analyses, which indicated that the differentially methylated genes play a role in activation of immune system processes. Finally, we used the Houseman correction with the Reinius dataset, a popular method to adjust for differences in cell type distributions between the exposed and unexposed groups of six cell types (B cells, granulocytes, monocytes, NK cells, CD4+ T cells and CD8+ T cells).^28^^,^^29^ This, reassuringly, showed no alterations in our top findings. The top signals still survived Bonferroni correction after cell type correction; however, the larger list of CpGs that survived FDR differed substantially (Supplementary Table S2, available as Supplementary data at *IJE* online). Consequently, the gene list that was used as input for the network and functional enrichment analysis was also different. Interestingly, the general pattern of results did not change, as we still observed that most enriched terms pointed towards positive regulation of particularly cell-mediated immune responses. Furthermore, the mediation results did not change as we observed significant mediation by the *GFI1* gene and not by any of the other genes, before and after cell type correction (mediation results before correction are not shown). This method was based on a reference dataset of whole blood samples from adult males, which have a different cell composition from cord blood, and this cell type correction did not account for more specific cell subtypes. However, currently this is the best option because no cord blood reference dataset exists and, even in cord blood, this reference-based cell type correction is the best method available as recently applied by Kile and colleagues.^27^

In contrast to an earlier study, which observed dose-dependency by maternal cotinine plasma levels,^17^ we did not find an effect of the number of cigarettes smoked per day. Joubert *et al.*^17^ found a dose-response relationship for two of the significant genes, but not for all top genes. Thus, a dose-response relationship could be expected for some genes but not for all. Another potential reason for the lack of a dose-response relationship in our data is our smaller sample size compared with the study of Joubert *et al*.

A potential limitation was the use of self-reported smoking behaviour during pregnancy. This may have caused underreporting of smoking behaviour and possibly could have resulted in an underestimation of the effects. In the GECKO Drenthe cohort, 14% of the mothers smoked during pregnancy. This is comparable to the prevalence of 7.6–13.2% found in The Netherlands in 2001–07^64^ and 12.3% in the USA.^65^ Furthermore, we observed results that were highly comparable to the study by Joubert *et al*., which measured smoking status objectively as plasma cotinine levels.^17^

We found support for our hypothesis that differential methylation mediates part of the effect of smoking on birthweight, but we could not be certain about the direction of causation in this observational study. One possibility is that methylation markers simply provided a better measure of smoking exposure than the self-reported smoking behaviour we used in our study. Such biomarkers would then also be expected to be associated with birthweight. However, the fact that only GFI1 showed significant association with birthweight and not, for example, the AHRR cg05575921 CpG showing the strongest EWAS signal, contradicted this explanation. Another possibility we could not entirely exclude is that retardation of fetal growth expressed as lower birthweight led to differential methylation rather than the other way around. However, we believe this is unlikely given the primary role of epigenetic mechanisms in orchestrating changes in gene expression during growth and development.

We acknowledge that the Baron and Kenny approach for mediation analysis has been criticized among others for its dependency on and sensitivity to measurement errors, misclassification and violation of model assumptions.^66^^,^^67^ However, the Infinium HumanMethylation450 BeadChip is a reliable instrument reflecting the state of the art in measurement of genome-wide DNA methylation.^68^ Moreover, mediation effects of three CpG sites were independently replicated in cord blood data from two other birth cohorts, in spite of presumably differential measurement errors between the three cohorts. Instability of methylation over time is an additional potentially important source of measurement error that could not be addressed by the cross-sectional design of our study, which only looked at differential methylation at birth. We backed up our mediation results from the Baron and Kenny approach with a more advanced statistical approach, and additionally applied causal mediation analysis to the three replicated CpGs in the GECKO cohort. This analysis uses a more general potential outcomes framework, can provide additional distribution-free estimates of the mediated effects and facilitates sensitivity analyses for the observed effects.^67^ Results of these analyses were in line with our Baron-Kenny results and Sobel tests (see Supplementary Note, available as Supplementary data at *IJE* online).

Previously, fathers who started smoking early were shown to have heavier sons,^69^ indicating a possible direct effect of paternal smoking on fetal programming through the sperm epigenome, which can affect embryogenesis.^70^^,^^71^ We did not explicitly test this possible direct effect in our study. However, only 39 (30%) of the fathers in the exposed group had smoked during pregnancy and, after excluding these children from the analysis, 83% of our top CpGs remained Bonferroni-significant. We also controlled for this possible paternal smoking effect in the study design, as we only included in the unexposed group those children whose mother and father did not smoke.

Our results suggested that *in utero* exposure to smoking could have an effect on selected methylation markers which may in turn affect later health outcomes in offspring. Our approach of testing the effects of intrauterine exposures on DNA methylation in the child may serve as a model that could be extended to other exposures. One example is fetal exposure to polycyclic aromatic hydrocarbons (PAHs), which has been linked to childhood obesity.^72^ PAHs are produced during incomplete combustion and are constituents not only of cigarette smoke but also of many other sources. Results of such studies may then provide guidance to future prevention efforts tailored to limit certain exposures for pregnant women with major potential impact on public health.

In conclusion, maternal tobacco smoking during pregnancy showed genome-wide methylation differences in 35 CpGs mapped to 10 genes measured in cord blood. Our results showed remarkable similarity to previous findings, confirming the robustness of the effects. Additionally, we observed a potentially mediating role of DNA methylation in the association between maternal smoking during pregnancy and birthweight of the offspring. We were able to replicate the mediating effect for three CpGs in *GFI1*, which confirmed and strengthened our findings. Finally, our network and enrichment analyses indicated that smoking in the mother may induce a cellular immune response in the fetus.

## Supplementary Data

Supplementary data are available at *IJE* online.

## Funding

This methylation project in GECKO was supported by the Biobanking and Biomolecular Research Infrastructure Netherlands [CP2011-19]. The GECKO Drenthe birth cohort was funded by an unrestricted grant of Hutchison Whampoa Ld, Hong Kong. The UK Medical Research Council and the Wellcome Trust [Grant no: 092 731] and the University of Bristol provide core support for ALSPAC. Funding for generation of DNA methylation data in ALSPAC was provided by the UK BBSRC [BB/I02575/1 and BB/I025263/1]. C.L.R. is supported by the MRC Integrative Epidemiology Unit (IEU) funded by the UK Medical Research Council [MC_UU_12013] and the University of Bristol. Funding for generation of DNA methylation data in ALSPAC was provided by the UK BBSRC [BB/I02575/1 and BB/I025263/1]. C.L.R. is partially supported by the ESRC [RES-060-23-0011]: ‘The biosocial archive: transforming lifecourse social research through the incorporation of epigenetic measures’. R.C.R. is funded by a Wellcome Trust 4-year PhD studentship [Grant Code: WT083431MF]. R.C.R. and C.L.R. are members of the MRC Integrative Epidemiology Unit (IEU) funded by the University of Bristol and the UK Medical Research Council [MC_UU_12013]. The Generation R Study is conducted by the Erasmus Medical Centre in close collaboration with the School of Law and Faculty of Social Sciences of the Erasmus University Rotterdam, the Municipal Health Service Rotterdam area, Rotterdam, the Rotterdam Homecare Foundation, Rotterdam and the Stichting Trombosedienst and Artsenlaboratorium Rijnmond (STAR), Rotterdam. The general design of Generation R Study is made possible by financial support from the Erasmus Medical Center, Rotterdam, the Erasmus University Rotterdam, the Netherlands Organization for Health Research and Development (ZonMw), The Netherlands Organisation for Scientific Research (NWO), the Ministry of Health, Welfare and Sport and the Ministry of Youth and Families. The generation and management of the Illumina 450 K methylation array data (EWAS data) for the Generation R Study was executed by the Human Genotyping Facility of the Genetic Laboratory of the Department of Internal Medicine, Erasmus MC, The Netherlands. The EWAS data were partially funded by The Netherlands Genomics Initiative (NGI)/ Netherlands Organization for Scientific Research (NWO) Netherlands Consortium for Healthy Aging (NCHA; project nr. 050-060-810), the Genetic Laboratory of the Department of Internal Medicine, Erasmus MC, The Netherlands Organization for Health Research and Development [VIDI 016.136.361] and the National Institutes of Health [1R01HL111108-01A1, 5R01NR013945-02]. The work of H.T. was supported by NWO-ZonMw Gravitation 2012 [BOO 024.001.003]. L.D. received a grant from the Lung Foundation Netherlands [no 3.2.12.089; 2012]. The study sponsors had no role in (i) the design and conduct of the study; ii) the collection, management, analysis and interpretation of the data; (iii) the preparation, review, or approval of the manuscript; or (iv) the decision to submit the manuscript for publication.

## Supplementary Material

Supplementary Data
