# Supplementary material for: DNA methylation mediates the effect of maternal smoking during pregnancy on birthweight of the offspring
Source: Int J Epidemiol. 2015 Apr 10;44(4):1224–37. doi: 10.1093/ije/dyv048 (PMC4588868; doi:10.1093/ije/dyv048)
Supplement: Supplementary Data [file supp_44_4_1224__index.html]

DNA methylation mediates the effect of maternal smoking during pregnancy on birthweight of the offspring — DNA methylation mediates the effect of maternal smoking during pregnancy on birthweight of the offspring — Supplementary Data 

# DNA methylation mediates the effect of maternal smoking during pregnancy on birthweight of the offspring

## Supplementary Data

files

**Files in this Data Supplement:**

- Supplementary Data - pdf file
