# Supplementary material for: DNA methylation mediates the effect of maternal smoking during pregnancy on birthweight of the offspring
Source: Int J Epidemiol. 2015 Apr 10;44(4):1224–37. doi: 10.1093/ije/dyv048 (PMC4588868; doi:10.1093/ije/dyv048)
Supplement: Supplementary Data [file supp_dyv048_Supplement_R3.pdf]

Supplemental material

DNA methylation mediates the effect of maternal smoking during pregnancy on birth weight of the offspring

Leanne K. Küpers[1], Xiaojing Xu[5], Soesma Jankipersadsing[2,3], Ahmad Vaez[1], Sacha la Bastide - van Gemert[1], Salome Scholtens[1,4], Ilja M. Nolte[1], Rebecca Richmond[6], Caroline L. Relton[6], Janine F. Felix[7], Liesbeth Duijts[7], Joyce B. van Meurs[7], Henning Tiemeier[7], Vincent W. Jaddoe[7], Xiaoling Wang[5], Eva Corpeleijn[1], Harold Snieder[1]

[1]Departments of Epidemiology, [2]Pulmonology and [3]Genetics, [4]LifeLines Cohort Study, University of Groningen, University Medical Center Groningen, Groningen, the Netherlands;

[5]Georgia Regents University, Augusta, Georgia, USA;

[6]University of Bristol, Bristol, United Kingdom;

[7]Erasmus MC, University Medical Center Rotterdam, the Netherlands

Contents

- Figure S1. Flowchart of selection procedure GECKO.
- ALSPAC and Generation R cohort descriptions.
- Table S1. Characteristics of children exposed and unexposed to maternal smoking, for replication cohorts ALSPAC and Generation R.
- Table S2. Methylation differences in GECKO for top CpGs in the crude, covariate-adjusted and completely-adjusted model including cell type composition (FDR<0.05).
- Table S3. Mediation results (Sobel P>0.05) for the indirect effect of maternal smoking on birth weight through methylation in GECKO.
- Table S4a-h. Interaction between covariates and individual CpGs.
- Table S5. Replication analysis for EWAS and mediation in the GFI1 CpGs in ALSPAC and Generation R.
- Supplementary note: Structural equation and causal mediation modelling.

N = 2874. Total number of participants in the GECKO Drenthe birth cohort.

N = 1565. Cord blood stored directly after birth (-80˚C).

N = 1404. Good DNA isolation (DNA concentration ≥ 50µg/ml).

N = 1118. After excluding premature newborns (≤37 weeks), twins, those with a mother with (gestational) diabetes and non Dutch newborns. And those with missing information on these variables.

N = 447. Newborns with information about maternal and paternal smoking during pregnancy (129 exposed to maternal smoking versus 318 unexposed to maternal and paternal smoking).

N = 258. Newborns selected for methylation analysis (129 exposed versus 129 unexposed).

N = 255. Final sample for methylation analysis after quality control (129 exposed versus 126 unexposed).

Figure S1. Flowchart of selection procedure GECKO.

ALSPAC cohort description

Design and study population

ALSPAC is a large, prospective cohort study based in the South West of England. 14,541 pregnant women resident in Avon, UK with expected dates of delivery 1st April 1991 to 31st December 1992 were recruited and detailed information has been collected on these women and their offspring at regular intervals[1,2]. The study website contains details of all the data that is available through a fully searchable data dictionary[3]. Written informed consent has been obtained for all ALSPAC participants. Ethical approval for the study was obtained from the ALSPAC Ethics and Law Committee and the Local Research Ethics Committees.

As part of the Accessible Resource for Integrated Epigenomic Studies (ARIES) project[4], the Infinium 450K BeadChip has been used to generate epigenetic data on 1,018 mother-offspring pairs in the ALSPAC cohort. The ARIES participants were selected based on availability of DNA samples at two time points for the mother (antenatal and at follow-up when the offspring were adolescents) and three time points for the offspring (neonatal, childhood (age 7) and adolescence (age 17)).

Of the 1,018 mother-offspring pairs in the ARIES project, 916 offspring had cord blood methylation data which successfully passed QC. Of these, we excluded twins, individuals with a gestational age <=37 weeks, children of mothers with diabetes during pregnancy and children missing any information on sex, gestational age, maternal age at birth, maternal pre-pregnancy BMI, maternal education, paternal smoking or birth weight, resulting in 678 complete cases for analysis.

Maternal smoking

Information on mothers' smoking status during pregnancy was obtained in questionnaires administered at 18 and 32 weeks gestation. Information was obtained about whether the mother smoked in each trimester of pregnancy and the number of cigarettes smoked on average per day. From these data, a dichotomous variable for sustained maternal smoking during pregnancy was derived. A mother was classified as a sustained smoker if she smoked in all three trimesters, smoked in the first and third trimester but not the second, or smoked in the second and third trimesters but not the first. The reference group consisted of mothers who had reported not smoking in all three trimesters. We excluded all individuals who smoked in one trimester only (i.e. not sustained) or who had missing information of smoking for two or more trimesters. Of those with missing information on one trimester, women were classified as a sustained smoker if they said they smoked in the other two trimesters.

Epigenome-wide association analyses

Cord blood samples were collected according to standard procedures. The DNA methylation wet-lab and pre-processing analyses were performed at the University of Bristol as part of the ARIES project. Following extraction, DNA was bisulfite converted using the Zymo EZ DNA MethylationTM kit (Zymo, Irvine, CA). Following conversion, genome-wide methylation status of over 485,000 CpG sites was measured using the Infinium HM450 Beadchip according to the standard protocol. The arrays were scanned using an Illumina iScan and initial quality review was assessed using GenomeStudio (version 2011.1).

Samples from all time points in ARIES were distributed across slides using a semi-random approach (sampling criteria were in place to ensure that all time points were represented on each array) to minimise the possibility of confounding by batch effects. In addition, during the data generation process a wide range of batch variables were recorded in a purpose-built laboratory information management system (LIMS). The main batch variable

was found to be the bisulphite conversion (BCD) plate number. Samples were converted in batches of 48 samples and each batch identified by a plate number.

The LIMS also reported quality control (QC) metrics from the standard control probes on the 450K BeadChip for each sample. Samples failing QC (average probe p-value >= 0.01) were repeated and if unsuccessful excluded from further analysis. As an additional QC step genotype probes were compared with SNP-chip data from the same individual to identify and remove any sample mismatches. For individuals with no genome-wide SNP data, samples were flagged if there was a sex-mismatch based on X-chromosome methylation.

In addition to these QC steps, probes that contained <95% of signals detectable above background signal (detection p-value <0.01) (N=7,938) were excluded from analysis. After excluding these probes, as well as control probes and probes on sex chromosomes, a total of 466,432 CpGs were included in the main analysis for cord blood methylation.

This resulted in 466,432 CpGs and 678 samples were analysed using Limma for EWAS and STATA for mediation analysis.

Methylation data were pre-processed in R (version 3.0.1), with background correction and subset quantile normalisation performed using the pipeline described by Touleimat and Tost[5].

Covariates

Variables considered as potential confounders in this analysis were offspring sex, gestational age, maternal age at birth, pre-pregnancy BMI, maternal education and paternal smoking. Gestational age and sex were recorded in the delivery room or abstracted from obstetric records and/or birth notifications. Gestational age was based on the date of the mother's last menstrual period, clinical records or ultrasounds. Maternal age at delivery was derived from date of birth, which was recorded at that time. At enrolment, the mother was asked to record her height and pre-pregnancy weight, from which BMI was calculated. Based on questionnaire responses, highest educational qualification for the mother was collapsed into a binary variable of low/middle education or university education. Information on partners' smoking during pregnancy was obtained from self-reports at 18 weeks gestation. Where self-reported data on partner smoking were not available, maternal reports were used. The bisulphite conversion batch for each sample was also included in the analysis in order to adjust for batch effects. Birth weight was considered as an outcome in this analysis. Infant birth weight was recorded in the delivery room or abstracted from obstetric records and/or birth notifications.

References

1. Boyd A, Golding J, Macleod J, et al. Cohort Profile: the 'children of the 90s'--the index offspring of the Avon Longitudinal Study of Parents and Children. *Int J Epidemiol* 2013; **42**: 111-27.
2. Fraser A, Macdonald-Wallis C, Tilling K, et al. Cohort Profile: the Avon Longitudinal Study of Parents and Children: ALSPAC mothers cohort. *Int J Epidemiol* 2013; **42**: 97-110.
3. ALSPAC. *Avon Longitudinal Study of Parents and Children*. [cited; Available from: http://www.bris.ac.uk/alspac/researchers/data-access/data-dictionary/
4. ARIES. *Accessible Resource for Integrated Epigenomic Studies*. [cited; Available from: http://www.ariesepigenomics.org.uk/ariesexplorer
5. Touleimat N, Tost J. Complete pipeline for Infinium((R)) Human Methylation 450K BeadChip data processing using subset quantile normalization for accurate DNA methylation estimation. *Epigenomics* 2012; **4**: 325-41.

Generation R cohort description

Design and study population

The Generation R Study is a population-based prospective cohort study from foetal life onwards in Rotterdam, the Netherlands. The study design has been described in detail elsewhere[1,2]. All children were born between April 2002 and January 2006 and form a largely prenatally enrolled birth cohort that is being followed until young adulthood. A total of 9778 mothers were included, most during pregnancy. Response rate at birth was 61%. Epigenome-wide association studies were done in 979 Caucasian children. The study protocol was approved by the Medical Ethical Committee of the Erasmus Medical Centre, Rotterdam. Written informed consent was obtained from all participants. The generation and management of EWAS data for the Generation R Study were done at the Genetic Laboratory of the Department of Internal Medicine, Erasmus MC, the Netherlands. The Generation R Study is conducted by the Erasmus Medical Center in close collaboration with the School of Law and Faculty of Social Sciences of the Erasmus University Rotterdam, the Municipal Health Service Rotterdam area, Rotterdam, the Rotterdam Homecare Foundation, Rotterdam and the Stichting Trombosedienst & Artsenlaboratorium Rijnmond (STAR-MDC), Rotterdam

Maternal smoking

Maternal prenatal smoking was assessed by questionnaire in early (<18 weeks gestational age), mid- (18-25 weeks gestational age) and late (>25 weeks gestational age) pregnancy. Women were asked whether and how much they had smoked. Sustained smoking was defined as continued smoking of >=1 cigarette per day throughout pregnancy. Women who quit smoking during pregnancy were not classified as sustained smokers and were not included in the sustained smoking analysis.

Epigenome-wide association analyses

DNA (500 ng per sample) was extracted from cord blood and underwent bisulfite conversion (EZ-96 DNA Methylation kit (Shallow), Zymo Research Corporation, Irvine, USA). Samples were plated onto 96-well plates in no specific order and Illumina Infinium HumanMethylation450 BeadChip (Illumina Inc., San Diego, USA) arrays were run.

Quality control was performed using the following criteria: Samples were excluded in case of low sample call rate (<99%), colour balance >3, low staining efficiency, poor extension efficiency, poor hybridization performance, low stripping efficiency after extension and poor bisulfite conversion. Based on this, we excluded of 7 samples (6 for low sample call rates, 1 for poor bisulfite conversion). In addition, 2 samples were excluded because of a sex mismatch and 1 sample because of a retracted informed consent, leaving 969 samples in the analysis.

Probes with a single nucleotide polymorphism in the single base extension site with a frequency of > 1% in the GoNLv4 reference panel were excluded, as were probes with non-optimal binding (non-mapping or mapping multiple times to either the normal or the bisulphite-converted genome), resulting in the exclusion of 49,564 probes, leaving a total of 436,013 probes in the analysis.

Data were normalized with DASES normalization using a pipeline adapted from that developed by Touleimat and Tost[3]. DASES consists of background adjustment, between-array normalization applied to type I and type II probes separately, and dye bias correction applied to type I and type II probes separately. DASES is based on the DASEN method

described by *Pidsley et al*, but adds the dye bias correction[4]. We calculated beta-values all CpG sites.

Cell type correction was applied using the reference-based Houseman method[5] in the minfi package[6] in R.

Covariates

Information on maternal age, parity, maternal education and paternal smoking was collected from questionnaires which were filled in by the mother at enrolment. Maternal body mass index (BMI, kg/m^2) was calculated from maternal height and weight, measured without shoes and heavy clothing at enrolment in early pregnancy. Information on child sex and gestational age was obtained from midwives' and obstetrician records. Maternal age, maternal BMI and gestational age were used as continuous covariates. Parity was categorized into 0 or >=1. Maternal education was categorized into lower (none, primary or secondary education) and higher (more than secondary education). Paternal smoking was categorized into smoking and non-smoking. Analyses were additionally adjusted for batch effects by adding plate number (11 categories) as a covariate.

References

1. Jaddoe VW, van Duijn CM, Franco OH, et al. The Generation R Study: design and cohort update 2012. *Eur J Epidemiol* 2012; **27**: 739-56.
2. Kruithof CJ, Kooijman MN, van Duijn CM, Franco OH, de Jongste JC, Klaver CC, Mackenbach JP, Moll HA, Raat H, Rings EH, Rivadeneira F, Steegers EA, Tiemeier H, Uitterlinden AG, Verhulst FC, Wolvius EB, Hofman A, Jaddoe VW. The Generation R Study: Biobank update 2015. *Eur J Epidemiol*. 2014;29:911-27.
3. Touleimat N, Tost J. Complete pipeline for Infinium((R)) Human Methylation 450K BeadChip data processing using subset quantile normalization for accurate DNA methylation estimation. *Epigenomics* 2012; **4**: 325-41.
4. Pidsley R, CC YW, Volta M, Lunnon K, Mill J, Schalkwyk LC. A data-driven approach to preprocessing Illumina 450K methylation array data. *BMC Genomics* 2013; **14**: 293.
5. Houseman EA, Accomando WP, Koestler DC, et al. DNA methylation arrays as surrogate measures of cell mixture distribution. *BMC Bioinformatics* 2012; **13**: 86.
6. Aryee MJ, Jaffe AE, Corrada-Bravo H, et al. Minfi: a flexible and comprehensive Bioconductor package for the analysis of Infinium DNA methylation microarrays. *Bioinformatics* 2014; **30**: 1363-9.

Table S1. Characteristics of children exposed and unexposed to maternal smoking, for replication cohorts ALSPAC and Generation R.

	ALSPAC			Generation R		
	**Unexposed (n=613)**	**Exposed (n=65)**	**$P_{difference}$**	**Unexposed (n=635)**	**Exposed (n=110)**	**$P_{difference}$**
Male	297 (48.4)	31 (47.7)	0.91	325 (51.2)	62 (56.4)	0.315
Birth weight	3531 ± 446	3359 ± 480	0.003	3608 ± 462	3395 ± 490	1.2E-05
Gestational age	39.9 ± 1.1	39.8 ± 1.3	0.73	40.3 ± 1.2	40.3 ± 1.1	0.733
Maternal age at child-birth	30.4 ± 4.2	28.1 ± 5.3	<0.001	31.9 ± 3.8	29.7 ± 5.4	9.1E-05
Maternal low/middle educational level	467 (76.2)	59 (90.8)	0.007	197 (31.0)	74 (67.3)	2.96E-13
Maternal pre-pregnancy BMI	22.8 ± 3.7	23.2 ± 3.9	0.42	24.3 ± 4.1	24.9 ± 4.8	0.205
Number of cigarettes smoked (in exposed group)	0	9 (2.5-26.5)	<0.001	-	-	-

Data shown as n (%) or mean ± SD. Except for number of cigarettes smoked: median (range).
P-values are given for independent samples t-test (continuous) or chi-square test (categorical).
Exposed group was defined as sustained smoking during pregnancy by mother. Unexposed group was defined as no smoking during pregnancy by mother nor by father.

Table S2. Methylation differences in GECKO for top CpGs in the crude, covariate-adjusted and completely-adjusted model including cell type composition (FDR<0.05).

		**Crude model CpG ~ smoking**		**Model incl. covariates CpG ~smoking + cov**		**Complete model CpG ~ smoking + cov + cell**	
**CpG**	**Closest gene**	**Methylation difference**	**P value**	**Methylation difference**	**P value**	**Methylation difference**	**P value**
cg05575921	*AHRR*	**-0.078**	**1.56E-31**	**-0.075**	**1.26E-27**	**-0.073**	**1.14E-25**
cg04180046	*MYO1G*	**0.060**	**2.86E-18**	**0.057**	**1.11E-15**	**0.056**	**1.10E-14**
cg09935388	*GFI1*	**-0.101**	**1.82E-15**	**-0.102**	**1.19E-14**	**-0.105**	**2.67E-14**
cg12876356	*GFI1*	**-0.110**	**4.26E-14**	**-0.110**	**6.17E-13**	**-0.107**	**1.79E-11**
cg18146737	*GFI1*	**-0.118**	**3.64E-13**	**-0.115**	**8.89E-12**	**-0.117**	**3.81E-11**
cg12803068	*MYO1G*	**0.076**	**6.44E-13**	**0.075**	**4.52E-12**	**0.077**	**1.79E-11**
cg18316974	*GFI1*	**-0.103**	**4.26E-12**	**-0.103**	**3.11E-11**	**-0.102**	**3.27E-10**
cg25949550	*CNTNAP2*	**-0.024**	**7.90E-12**	**-0.024**	**4.09E-11**	**-0.022**	**9.84E-09**
cg21611682	*LRP5*	**-0.023**	**3.75E-11**	**-0.022**	**5.93E-10**	**-0.021**	**2.83E-10**
cg22132788	*MYO1G*	**0.050**	**3.95E-11**	**0.051**	**9.22E-11**	**0.053**	**1.57E-10**
cg14179389	*GFI1*	**-0.058**	**5.29E-11**	**-0.061**	**3.81E-11**	**-0.061**	**1.76E-11**
cg21161138	*AHRR*	**-0.032**	**5.60E-11**	**-0.032**	**1.22E-10**	**-0.024**	**6.18E-08**
cg05549655	*CYP1A1*	**0.041**	**1.53E-10**	**0.039**	**3.80E-11**	**0.036**	**3.20E-09**
cg11429111	-	**0.044**	**1.55E-10**	**0.044**	**7.68E-11**	**0.048**	**7.17E-12**
cg23067299	*AHRR*	**0.037**	**1.63E-10**	**0.038**	**1.01E-10**	**0.038**	**2.66E-10**
cg09662411	*GFI1*	**-0.065**	**8.29E-10**	**-0.066**	**2.98E-09**	**-0.066**	**9.55E-09**
cg19089201	*MYO1G*	**0.035**	**1.40E-09**	**0.035**	**3.42E-09**	**0.037**	**3.53E-09**
cg11924019	*CYP1A1*	**0.038**	**6.36E-09**	**0.035**	**5.15E-09**	**0.036**	**9.04E-09**
cg25737891	-	**0.029**	**1.27E-08**	**0.027**	**3.67E-07**	0.016	1.14E-03
cg22549041	*CYP1A1*	**0.055**	**2.24E-08**	**0.052**	**5.25E-09**	**0.052**	**1.53E-08**
cg01952185	-	**0.040**	**2.59E-08**	**0.039**	**1.58E-08**	**0.047**	**3.32E-11**
cg16547579	*SLC23A2*	**-0.034**	**3.28E-08**	**-0.032**	**6.35E-07**	-0.025	2.21E-05
cg03243902	*TRAPPC9*	**-0.019**	**5.02E-08**	**-0.020**	**3.43E-08**	-0.011	3.49E-04
cg23916896	*AHRR*	**-0.032**	**5.91E-08**	**-0.029**	**2.50E-06**	-0.026	2.77E-05
cg18092474	*CYP1A1*	**0.049**	**9.84E-08**	**0.046**	**4.85E-08**	**0.047**	**9.24E-08**
cg00253658	-	**0.040**	**1.27E-07**	**0.036**	**1.51E-06**	0.033	2.74E-05
cg23928512	*ASPSCR1*	**-0.022**	**1.37E-07**	**-0.019**	**9.34E-07**	-0.017	1.96E-05
cg06338710	*GFI1*	**-0.065**	**1.75E-07**	**-0.063**	**9.43E-07**	-0.059	7.58E-06
cg15507334	*FRMD4A*	**0.026**	**1.92E-07**	**0.028**	**3.84E-10**	**0.028**	**2.90E-09**
cg26703534	*AHRR*	**-0.018**	**3.01E-07**	-0.017	6.18E-06	-0.015	9.35E-05
cg13834112	-	**0.030**	**3.11E-07**	0.027	5.32E-06	**0.028**	**4.54E-06**
cg04535902	*GFI1*	**-0.057**	**3.26E-07**	**-0.058**	**5.04E-07**	**-0.057**	**1.77E-06**
cg03384915	*SIN3B*	**-0.018**	**3.68E-07**	-0.017	1.22E-06	-0.015	9.88E-06
cg22937882	*AHRR*	**0.015**	**4.18E-07**	**0.016**	**1.56E-07**	**0.016**	**2.23E-07**
cg24691891	*PHC2*	**-0.019**	**5.15E-07**	-0.018	3.31E-06	-0.008	3.54E-03
cg14449069	-	**-0.037**	**5.75E-07**	-0.034	5.74E-06	-0.034	1.21E-05
cg06060703	*CCDC78*	**-0.021**	**7.21E-07**	-0.020	7.81E-06	-0.008	2.16E-02
cg20244340	*SLC24A3*	**-0.022**	**9.71E-07**	**-0.023**	**9.96E-07**	-0.022	1.14E-05
cg14866547	*EYA4*	**-0.023**	**1.00E-06**	-0.021	1.76E-05	-0.011	1.56E-02
cg11902777	*AHRR*	**-0.013**	**1.13E-06**	-0.012	1.47E-05	-0.009	1.47E-03
cg23275914	*TTLL10*	**-0.019**	**1.14E-06**	-0.017	3.95E-05	-0.007	2.47E-02
cg18479322	*PACRG*	**-0.018**	**1.18E-06**	-0.019	7.84E-07	-0.013	5.03E-04
cg27180298	-	**-0.017**	**1.50E-06**	-0.016	8.26E-06	-0.012	1.06E-03
cg21322848	*SEMA6D*	**-0.020**	**1.68E-06**	**-0.021**	**1.85E-06**	-0.018	5.52E-05
cg03413253	-	**-0.026**	**1.78E-06**	**-0.027**	**3.43E-06**	-0.016	3.50E-03
cg02252828	*MTMR9L*	**-0.019**	**1.99E-06**	**-0.020**	**1.09E-06**	-0.015	3.09E-04
cg25152368	*PRDM16*	**-0.006**	**2.46E-06**	**-0.007**	**1.67E-06**	-0.007	7.66E-06
cg20914572	*PRRT1*	**0.016**	**2.52E-06**	0.015	1.05E-05	0.012	3.73E-04
cg18425731	*ERMN*	**-0.014**	**2.53E-06**	-0.014	8.44E-06	-0.009	3.06E-03
cg04923840	*KCNK2*	**-0.027**	**2.80E-06**	**-0.027**	**4.32E-06**	-0.015	4.97E-03
cg11207515	*CNTNAP2*	**-0.044**	**2.97E-06**	**-0.048**	**1.44E-06**	-0.030	1.23E-03
cg12322877	*ASPSCR1*	**-0.022**	**3.07E-06**	-0.019	3.17E-05	-0.018	1.33E-04
cg10146692	*BOP1*	**-0.015**	**3.33E-06**	-0.014	3.49E-05	-0.009	3.52E-03

cg02752334	*PRKAG2*	**-0.020**	**3.68E-06**	-0.018	5.31E-05	-0.008	2.94E-02
cg11823551	-	**-0.016**	**3.71E-06**	-0.014	9.49E-05	-0.008	7.02E-03
cg04915173	*CDC123*	**-0.026**	**4.10E-06**	**-0.029**	**4.75E-07**	-0.019	4.61E-04
cg00415333	*IL22RA2*	**-0.022**	**4.16E-06**	-0.020	5.94E-05	-0.013	4.66E-03
cg18578939	-	**-0.011**	**4.38E-06**	**-0.011**	**4.15E-06**	-0.007	9.88E-04
cg13570656	*CYP1A1*	**0.043**	**4.40E-06**	**0.039**	**4.17E-06**	0.039	1.29E-05
cg06757863	*CNTN5*	**-0.015**	**4.55E-06**	-0.013	9.45E-05	-0.012	8.82E-04
cg12101586	*CYP1A1*	**0.041**	**5.09E-06**	**0.037**	**5.40E-06**	**0.040**	**2.11E-06**
cg04398416	-	**-0.052**	**5.13E-06**	-0.053	1.18E-05	-0.038	2.04E-03
cg26534812	-	**-0.016**	**5.14E-06**	-0.014	6.68E-05	-0.008	2.35E-03
cg08606254	*AHRR*	**0.023**	**5.22E-06**	**0.024**	**2.84E-06**	0.023	2.26E-05
cg01866178	-	**-0.027**	**5.31E-06**	-0.027	1.44E-05	-0.014	1.45E-02
cg00671823	*GTF3C1*	**-0.013**	**5.65E-06**	-0.014	1.52E-05	-0.011	4.26E-04
cg12629909	*PPT2*	**-0.015**	**5.75E-06**	-0.015	2.33E-05	-0.010	2.65E-03
cg23680900	*CYP1A1*	**0.014**	**5.97E-06**	0.013	5.31E-05	**0.015**	**5.01E-06**
cg25918833	*OR51E2*	**-0.024**	**6.65E-06**	-0.025	1.06E-05	-0.013	1.11E-02
cg23249717	-	**-0.013**	**6.68E-06**	-0.012	7.03E-05	-0.008	5.99E-03
cg11650584	-	**-0.010**	**6.84E-06**	-0.009	1.37E-04	-0.006	1.43E-02
cg01945508	*STX2*	**-0.017**	**7.06E-06**	-0.018	7.90E-06	-0.013	1.62E-03
cg05556923	*PDGFA*	**-0.008**	**7.13E-06**	-0.008	3.35E-05	-0.007	4.68E-04
cg03631294	*CD79B*	**-0.016**	**7.15E-06**	-0.015	3.04E-05	-0.006	4.67E-02
cg25189904	*GNG12*	**-0.024**	**7.36E-06**	-0.021	6.67E-05	-0.019	3.42E-04
cg01970407	*AHRR*	0.022	8.90E-06	**0.025**	**2.04E-07**	**0.023**	**4.07E-06**
cg23335299	-	0.033	1.50E-05	**0.033**	**3.08E-07**	0.025	4.18E-05
cg04358214	*C16orf70*	0.032	9.19E-05	**0.038**	**6.45E-07**	0.034	2.14E-05
cg25464840	*FRMD4A*	0.022	2.15E-05	**0.023**	**9.21E-07**	**0.025**	**3.07E-07**
cg18700329	-	-0.029	1.16E-05	**-0.033**	**1.31E-06**	-0.025	3.16E-04
cg15424989	*GATA6*	-0.022	2.37E-05	**-0.026**	**1.75E-06**	-0.019	4.53E-04
cg13916874	-	-0.020	1.20E-05	**-0.021**	**1.93E-06**	-0.016	4.29E-04
cg05343480	*ZNF467*	-0.011	1.89E-05	**-0.012**	**3.03E-06**	-0.008	1.00E-03
cg14126777	*SPIRE1*	-0.028	1.97E-05	**-0.031**	**3.06E-06**	-0.021	7.95E-04
cg16398965	-	-0.016	2.28E-05	**-0.018**	**3.57E-06**	-0.014	2.34E-04
cg08930944	*OR12D2*	-0.025	5.02E-05	**-0.029**	**3.79E-06**	-0.020	9.66E-04
cg24159436	*PLCL2*	0.023	5.98E-05	**0.026**	**4.03E-06**	**0.028**	**1.10E-06**
cg08993681	-	-0.011	3.55E-05	**-0.013**	**4.26E-06**	-0.009	1.07E-03
cg15636519	*STAT4*	0.023	1.52E-05	**0.025**	**4.81E-06**	0.017	1.92E-03
cg16136238	*IGFBP7*	-0.025	2.12E-05	**-0.028**	**5.16E-06**	-0.021	9.12E-04
cg04111467	*IQCG*	-0.015	1.21E-05	**-0.016**	**6.04E-06**	-0.012	1.01E-03
cg08421910	*C6orf105*	0.026	2.37E-05	**0.028**	**6.58E-06**	0.019	5.09E-04
cg20308204	*RFPL3*	-0.011	7.94E-06	**-0.011**	**6.75E-06**	-0.009	3.57E-04
cg13822849	*OLFM1*	0.021	3.93E-05	**0.020**	**6.79E-06**	0.020	2.06E-05
cg04329870	*CD8B*	-0.018	1.04E-05	**-0.019**	**6.90E-06**	-0.013	5.75E-04
cg14817490	*AHRR*	-0.019	2.38E-03	-0.023	3.14E-04	**-0.03**	**3.98E-08**
cg11813497	*FRMD4A*	0.023	9.00E-05	0.026	7.50E-06	**0.028**	**4.01E-06**
cg17292337	-	-0.082	3.00E-05	-0.089	1.34E-05	**-0.098**	**5.14E-06**
cg01264106	*LGALS1*	0.019	3.67E-05	0.019	1.04E-04	**0.02**	**5.78E-06**
cg10399789	*GFI1*	-0.044	1.42E-05	-0.046	1.58E-05	**-0.049**	**7.48E-06**

Covariates: plate, sex, gestational age, maternal age, education and BMI.

Cell: cell type composition.

Bold: FDR<0.05

Table S3. Mediation results (Sobel P>0.05) for the indirect effect of maternal smoking on birth weight through methylation in GECKO.

	**Smoking to birth weight BW = smoking + covariates**				**Full model BW = smoking + CpG + covariates**						
	**β_c**	**SE_c**	**P_c**	**R-square**	**$\beta_{c'}$**	**β_b**	**SE_b**	**P_b**	**Difference in betas (β_c - $\beta_{c'}$)**	**Mediation percentage ((β_c - $\beta_{c'}$) / β_c)**	**Sobel P value**
AHRR											
cg05575921	-264.3	59.4	1.3E-05	0.307	-202.0	845.7	621.8	0.175	-62.3g	23.6%	0.1764
cg23067299	-264.3	59.4	1.3E-05	0.307	-280.2	426.4	674.1	0.528	15.9g	-6.0%	0.5292
cg14817490	-264.3	59.4	1.3E-05	0.307	-251.7	536.0	590.0	0.365	-12.6g	4.8%	0.3767
cg21161138	-264.3	59.4	1.3E-05	0.307	-269.6	-171.4	804.2	0.831	5.3g	-2.0%	0.8313
cg22937882	-264.3	59.4	1.3E-05	0.307	-243.6	-1298.1	1286.1	0.314	-20.7g	7.8%	0.3213
cg01970407	-264.3	59.4	1.3E-05	0.307	-250.7	-531.4	796.6	0.505	-13.6g	5.1%	0.5082
MYO1G											
cg04180046	-264.3	59.4	1.3E-05	0.307	-242.0	-394.4	564.9	0.486	-22.3g	8.4%	0.4867
cg12803068	-264.3	59.4	1.3E-05	0.307	-261.6	-35.7	363.6	0.922	-2.7g	1.0%	0.9496
cg22132788	-264.3	59.4	1.3E-05	0.307	-268.0	73.4	497.0	0.883	3.7g	-1.4%	0.8826
cg19089201	-264.3	59.4	1.3E-05	0.307	-272.7	240.9	657.1	0.714	8.4g	-3.2%	0.7144
CYP1A1											
cg05549655	-264.3	59.4	1.3E-05	0.307	-247.7	-425.3	661.1	0.521	-16.6g	6.3%	0.5220
cg11924019	-264.3	59.4	1.3E-05	0.307	-265.7	42.7	652.5	0.948	1.4g	-0.5%	0.9478
cg22549041	-264.3	59.4	1.3E-05	0.307	-251.3	-249.9	434.3	0.566	-13.0g	4.9%	0.5669
cg18092474	-264.3	59.4	1.3E-05	0.307	-259.4	-106.8	464.1	0.818	-4.9g	1.9%	0.8181
cg12101586	-264.3	59.4	1.3E-05	0.307	-249.4	-407.2	474.6	0.392	-14.9g	5.6%	0.3989
cg23680900	-264.3	59.4	1.3E-05	0.307	-271.4	549.9	1194.3	0.646	7.1g	-2.7%	0.6471
CNTNAP2											
cg25949550	-264.3	59.4	1.3E-05	0.307	-220.1	1820.5	1058.3	0.087	-44.2g	16.7%	0.098
FRMD4A											
cg15507334	-264.3	59.4	1.3E-05	0.307	-230.8	-1206.9	875.4	0.169	-33.5g	12.7%	0.1762
cg25464840	-264.3	59.4	1.3E-05	0.307	-240.9	-1013.4	825.3	0.221	-23.4g	8.9%	0.2355
cg11813497	-264.3	59.4	1.3E-05	0.307	-256.3	-308.2	668.7	0.645	-8.0g	3.0%	0.6467

LRP5											
cg21611682	-264.3	59.4	1.3E-05	0.307	-229.2	1619.8	1107.0	0.145	-35.1g	13.3%	0.1513
PLCL2											
cg24159436	-264.3	59.4	1.3E-05	0.307	-263.7	-23.5	698.0	0.973	-0.6g	0.2%	0.9731
LGALS1											
cg01264106	-264.3	59.4	1.3E-05	0.307	-245.3	-1018.4	793.5	0.201	-19.0g	7.2%	0.2240
-											
cg13834112	-264.3	59.4	1.3E-05	0.307	-236.0	-1038.7	634.5	0.103	-28.3g	10.7%	0.1240
cg17292337	-264.3	59.4	1.3E-05	0.307	-248.8	175.4	187.8	0.351	-15.5g	5.9%	0.3609

Covariates: plate, sex, gestational age, maternal age, maternal education, maternal BMI and cell type composition.

Sobel test statistic $Z = \beta_c - \beta_{c'}$ / SE, where $SE = \sqrt{(\beta_a^2 * SE_b^2 + \beta_b^2 * SE_a^2)}$

The coefficients βc and βc' can be interpreted as the amount of grams lower birth weight for smoking versus non-smoking mothers in the "smoking to birth weight" and full model, respectively. βb represents the effect of methylation level (coded as a proportion between 0-1) on birth weight. For cg09935388 this means that an increase of 100% in methylation level is associated with a 1190.4g higher birth weight. For extra information on the betas, see Figure 1.

Table S4a. Interaction between covariates and cg09935388

	Beta	*SE*	*P*
**Smoking**	-159.8	690.7	0.817
**Gender**	272.5	592.8	0.646
**Gestational age**	220.8	207.8	0.289
**Maternal educational level**	-289.9	636.2	0.649
**maternal pre-pregnancy BMI**	2.2	64.6	0.973
**Maternal age**	30.9	71.9	0.668
**Batch1**	204.2	626.8	0.745
**Batch2**	-844.7	584.1	0.149

Table S4b. Interaction between covariates and cg14179389

	Beta	*SE*	*P*
**Smoking**	617.0	974.4	0.527
**Gender**	315.6	927.0	0.734
**Gestational age**	281.3	308.1	0.362
**Maternal educational level**	104.5	938.0	0.911
**maternal pre-pregnancy BMI**	-5.0	111.3	0.964
**Maternal age**	15.0	113.6	0.895
**Batch1**	292.3	938.9	0.756
**Batch2**	-167.4	893.6	0.852

Table S4c. Interaction between covariates and cg12876356

	Beta	*SE*	*P*
**Smoking**	125.1	579.2	0.829
**Gender**	225.2	515.2	0.662
**Gestational age**	69.3	193.1	0.720
**Maternal educational level**	-914.4	558.9	0.103
**maternal pre-pregnancy BMI**	75.7	60.1	0.209
**Maternal age**	4.5	63.5	0.943
**Batch1**	-208.9	537.9	0.698
**Batch2**	-444.9	516.7	0.390

Table S4d. Interaction between covariates and cg18146737

	Beta	*SE*	*P*
**Smoking**	234.8	514.4	0.648
**Gender**	29.4	466.3	0.950
**Gestational age**	71.8	179.7	0.690
**Maternal educational level**	-815.9	515.1	0.114
**maternal pre-pregnancy BMI**	73.0	53.0	0.170
**Maternal age**	9.7	59.9	0.872
**Batch1**	-163.6	479.7	0.733
**Batch2**	-491.9	466.5	0.293

Table S4e. Interaction between covariates and cg18316974

	Beta	*SE*	*P*
**Smoking**	30.7	579.7	0.958
**Gender**	-22.6	519.8	0.965
**Gestational age**	79.3	200.4	0.693
**Maternal educational level**	-797.5	546.3	0.146
**maternal pre-pregnancy BMI**	76.1	60.9	0.213
**Maternal age**	11.8	64.1	0.855
**Batch1**	-383.5	548.2	0.485
**Batch2**	-559.1	514.1	0.278

Table S4f. Interaction between covariates and cg09662411

	Beta	*SE*	*P*
**Smoking**	-22.9	791.0	0.977
**Gender**	321.7	737.7	0.663
**Gestational age**	-39.8	288.3	0.890
**Maternal educational level**	-1368.2	792.0	0.085
**maternal pre-pregnancy BMI**	111.9	86.4	0.196
**Maternal age**	51.7	94.4	0.584
**Batch1**	-823.8	750.7	0.273
**Batch2**	-231.0	744.6	0.757

Table S4g. Interaction between covariates and cg04535902

	Beta	*SE*	*P*
**Smoking**	-570.7	753.0	0.449
**Gender**	444.5	709.7	0.532
**Gestational age**	-21.8	267.0	0.935
**Maternal educational level**	-320.5	758.8	0.673
**maternal pre-pregnancy BMI**	75.7	87.3	0.387
**Maternal age**	13.4	85.6	0.876
**Batch1**	-565.4	785.8	0.472
**Batch2**	-776.6	704.1	0.271

Table S4h. Interaction between covariates and cg10399789

	Beta	*SE*	*P*
**Smoking**	-50.1	824.7	0.952
**Gender**	1002.2	805.5	0.215
**Gestational age**	-21.5	308.0	0.944
**Maternal educational level**	-169.8	845.7	0.841
**maternal pre-pregnancy BMI**	127.9	93.7	0.173
**Maternal age**	40.0	108.3	0.712
**Batch1**	-1510.5	834.7	0.072
**Batch2**	18.2	799.2	0.982

Table S5. Replication analysis for EWAS and mediation in the *GFI1* CpGs in ALSPAC and Generation R.

	EWAS		***Mediation analysis***													
			Smoking to birth weight<br>BW = smoking + covariates				*Smoking to methylation*<br>CpG = smoking + covariates			*Full model*<br>BW = smoking + CpG + covariates				*Mediation*		
	**Methylation difference**	**P**	**β_c**	**SE_c**	**P_c**	**R^2**	**β_a**	**SE_a**	**P_a**	**$\beta_{c'}$**	**β_b**	**SE_b**	**P_b**	**Δ betas (βc - $\beta c'$)**	**Mediation % ((β_c - $\beta_{c'}$) / β_c)**	**Sobel P**
ALSPAC																
cg09935388	-0.160	1.3E-09	-178	59.7	0.0030	0.01	-0.14	0.03	5.0E-08	-162.9	104.9	87.4	0.23	-15.1g	8.5%	0.245
cg14179389	-0.068	1.9E-07	-178	59.7	0.0030	0.01	-0.07	0.01	3.4E-07	-162.8	227.0	175.0	0.20	-15.2g	8.5%	0.202
cg12876356	-0.137	2.0E-08	-178	59.7	0.0030	0.01	-0.18	0.03	1.2E-07	-153.4	139.9	69.4	0.04	-24.6g	13.8%	0.056
cg18146737	-0.137	2.7E-09	-178	59.7	0.0030	0.01	-0.14	0.02	2.7E-09	-168.6	61.9	100.7	0.54	-9.4g	5.3%	0.540
cg18316974	-0.092	4.2E-05	-178	59.7	0.0030	0.01	-0.09	0.02	5.3E-05	-186.1	-87.8	102.4	0.39	8.1g	-4.6%	0.400
cg09662411	-0.149	2.5E-08	-178	59.7	0.0030	0.01	-0.13	0.02	8.2E-07	-176.8	9.4	84.6	0.91	-1.2g	0.7%	0.912
cg04535902	-0.092	6.5E-03	-178	59.7	0.0030	0.01	-0.09	0.03	6.0E-03	-181.6	-37.4	67.6	0.58	3.6g	-2.0%	0.586
cg10399789	-0.115	7.8E-04	-178	59.7	0.0030	0.01	-0.11	0.03	2.0E-03	-173.8	39.4	66.5	0.55	-4.2g	2.4%	0.559
GenerationR																
**cg09935388**	**-0.090**	**1.7E-12**	**-177**	**48.1**	**0.0003**	**0.219**	**-0.087**	**0.012**	**3.5E-12**	**-117.1**	**458.3**	**136.2**	**0.001**	**-60.1g**	33.9%	**0.002**
cg14179389	-0.062	2.3E-11	-177	48.1	0.0003	0.219	-0.060	0.009	2.1E-11	-134.1	378.6	190.1	0.047	-43.1g	24.3%	0.056
**cg12876356**	**-0.073**	**7.1E-09**	**-177**	**48.1**	**0.0003**	**0.219**	**-0.073**	**0.012**	**2.3E-09**	**-134.0**	**311.0**	**138.2**	**0.025**	**-43.2g**	24.4%	**0.035**
cg18146737	-0.081	1.1E-07	-177	48.1	0.0003	0.219	-0.081	0.015	4.4E-08	-138.6	224.3	114.7	0.051	-38.6g	21.8%	0.066
cg18316974	-0.048	3.6E-04	-177	48.1	0.0003	0.219	-0.049	0.013	1.5E-04	-149.3	151.7	129.1	0.240	-27.9g	15.7%	0.262
cg09662411	-0.038	2.8E-05	-177	48.1	0.0003	0.219	-0.039	0.009	1.3E-05	-143.3	342.0	187.6	0.069	-33.9g	19.1%	0.093
cg04535902	-0.009	3.3E-01	-177	48.1	0.0003	0.219	-0.013	0.009	1.5E-01	-159.4	-209.1	186.4	0.262	-17.8g	10.0%	0.376
cg10399789	-0.022	4.0E-02	-177	48.1	0.0003	0.219	-0.025	0.011	1.9E-02	-155.0	68.6	158	0.664	-22.2g	12.5%	0.670

Covariates: plate, sex, gestational age, maternal age, maternal education, maternal BMI (and cell type composition, only for *smoking to methylation*).

Sobel test statistic $Z = \beta_c - \beta_{c'} / SE$, where $SE = \sqrt{(\beta_a^2 * SE_b^2 + \beta_b^2 * SE_a^2)}$

Bold: Significant mediation (Sobel P<0.05).

The coefficients βc and βc' can be interpreted as the amount of grams lower birth weight for smoking versus non-smoking mothers in the "smoking to birth weight" and full model, respectively. βb represents the effect of methylation level (coded as a proportion between 0-1) on birth weight. For cg09935388 this means that an increase of 100% in methylation level is associated with a 1190.4g higher birth weight. For extra information on the betas, see Figure 1.

Supplementary Note: Structural equation and causal mediation modelling

Estimating mediation in structural equation models (GECKO)

We built identical structural equation models containing all background variables and the maternal smoking-CpG-birth weight mediation structure for the three CpGs that showed replication in the meta-analysis. The existence and direction of the various effects between the variables in these models were chosen based on logical reasoning, theory and previously established results. Model fit for all three models was evaluated using Pearson's χ^2 test statistic and χ^2/df. The null hypothesis of the χ^2 test states that the population covariance matrix of the measured variables is equal to the covariance matrix of the model, with $\chi^2/df<2$ indicating a good model fit. Estimations of regression coefficients and standard errors are given. We used the sem library[1] in R, version 3.1.1.

All three models had good model fit ($P>0.05$ and $\chi^2/df<2$), and in each model the regression coefficients for the three paths in the mediation-structure were significant ($P<0.05$).

Model fit of the three structural equation models:

SEM with CpG:	χ^2	df	p	χ^2/df
**cg09935388**	17.3	13	0.184	1.33
**cg14179389**	21.8	13	0.059	1.68
**cg12876356**	16.2	13	0.238	1.25

Structural Equation Model 1 - cg09935388

Path	Estimate	SE	z value	Pr(>|z|)
education mother -> BMI mother	-1.360	0.582	-2.340	1.95E-02
education mother -> age mother	2.080	0.555	3.740	1.82E-04
education mother -> gestational age	0.411	0.178	2.310	2.08E-02
education mother -> maternal smoking	-0.300	0.065	-4.620	3.84E-06
gender -> birth weight	164.0	54.1	3.030	2.45E-03
BMI mother -> birth weight	10.60	6.29	1.680	9.34E-02
BMI mother -> gestational age	0.048	0.018	2.710	6.65E-03
age mother -> birth weight	7.84	6.42	1.220	2.22E-01
age mother -> gestational age	-0.007	0.019	-0.365	7.15E-01
gestational age -> birth weight	181.3	21.9	8.270	1.30E-16
maternal smoking -> gestational age	-0.100	0.159	-0.624	5.32E-01
maternal smoking -> birth weight	-137.3	61.5	-2.230	2.55E-02
maternal smoking -> cg09935388	-0.101	0.012	-8.460	2.67E-15
cg09935388 -> birth weight	1179	284	4.160	3.24E-05

Structural Equation Model 2 - cg14179389

Path	Estimate	SE	z value	Pr(>|z|)
education mother -> BMI mother	-1.36	0.58	-2.34	1.95E-02
education mother -> age mother	2.08	0.56	3.74	1.82E-04
education mother -> gestational age	0.411	0.178	2.31	2.08E-02
education mother -> maternal smoking	-0.30	0.06	-4.62	3.84E-06
gender -> birth weight	181.10	55.40	3.27	1.09E-03
BMI mother -> birth weight	11.80	6.44	1.82	6.82E-02
BMI mother -> gestational age	0.0483	0.0178	2.71	6.65E-03
age mother -> birth weight	8.32	6.58	1.27	2.06E-01
age mother -> gestational age	-0.0068	0.019	-0.37	7.15E-01
gestational age -> birth weight	181.5	22.4	8.09	6.09E-16
maternal smoking -> gestational age	-0.0995	0.159	-0.62	5.32E-01
materal smoking -> birth weight	-207.8	60.5	-3.43	5.93E-04
maternal smoking -> cg14179389	-0.06	0.01	-6.84	7.88E-12
cg14179389 -> birth weight	852.1	408.7	2.09	3.71E-02

Structural Equation Model 3 - cg12876356

Path	Estimate	SE	z value	Pr(>|z|)
education mother -> BMI mother	-1.360	0.582	-2.34	1.95E-02
education mother -> age mother	2.080	0.555	3.74	1.82E-04
education mother -> gestational age	0.411	0.178	2.31	2.08E-02
education mother -> maternal smoking	-0.300	0.065	-4.62	3.84E-06
gender -> birth weight	170.5	54.3	3.13	1.69E-03
BMI mother -> birth weight	10.20	6.31	1.61	1.07E-01
BMI mother -> gestational age	0.048	0.018	2.71	6.65E-03
age mother -> birth weight	7.40	6.44	1.15	2.51E-01
age mother -> gestational age	-0.007	0.019	-0.37	7.15E-01
gestational age -> birth weight	179.9	22.0	8.18	2.74E-16
maternal smoking -> gestational age	-0.100	0.159	-0.62	5.32E-01
maternal smoking -> birth weight	-150.10	60.90	-2.46	1.37E-02
maternal smoking -> cg12876356	-0.110	0.014	-7.98	1.43E-15
cg12876356 -> birth weight	969.6	245.9	3.94	8.03E-05

Estimating mediation using causal mediation analysis (GECKO)

For each CpG we fitted both outcome and mediator models: a linear regression model for *CpG* given *maternal smoking* and all covariates (*gender, maternal educational level, maternal BMI, maternal age, gestational age*) and a linear regression model for *birth weight* given *CpG, maternal smoking* and the covariates respectively. We then used the general approach to causal mediation analysis as described by Imai et al.[2] to estimate mediation. Model parameters were estimated by simulation, based on their approximate asymptotic distribution and computing the causal mediation effects for each parameter draw. Results are given in the Quasi-Bayesian table. Alternatively, an approach based on nonparametric bootstrap was also applied which created a bootstrap distribution of the mediated and direct effects. Estimations of the effects and the CI's based on the percentiles of this distribution are given in the Nonparametric Bootstrap table. We used the mediate library[3] in R, version 3.1.1.

Effect sizes and p-values of the mediated effects were similar to the results from the Baron and Kenny approach and Sobel tests.

Quasi-Bayesian Confidence Intervals (simulations = 1000)

Model	ACME [95% CI]	P value	ADE [95% CI]	P value	Proportion mediated [95% CI]	P value
**cg09935388**	-119.5 [-189.7;-58.7]	<0.005	-143.3 [-274.9;-18.0]	0.02	0.456 [0.200;0.894]	<0.005
**cg14179389**	-51.6 [-108.9;2.15]	0.06	-211.0 [-333.7;-83.6]	<0.005	0.199 [-0.009;0.518]	0.06
**cg1287635**	-106.9 [-174.6;-51.6]	<0.005	-155.9 [-280.3;-24.3]	0.02	0.404 [0.190;0.842]	<0.005

ACME = averaged causal mediated effect. ADE = averaged direct effect

Nonparametric Bootstrap Confidence Intervals, with percentile method (simulations = 1000)

Model	ACME [95% CI]	P value	ADE [95% CI]	P value	Proportion mediated [95% CI]	P value
**cg09935388**	-120.2 [-185.4;-59.6]	<0.005	-143.1 [-265.8;-13.2]	0.03	0.457 [0.221;0.918]	<0.005
**cg14179389**	-51.3 [-108.9;-2.04]	0.05	-212.1 [-342.4;-81.9]	<0.005	0.195 [0.008;0.504]	0.05
**cg1287635**	-106.6 [-172.5;-48.7]	<0.005	-156.8 [-283.1;-38.3]	0.01	0.405 [0.17;0.767]	<0.005

ACME = averaged causal mediated effect. ADE = averaged direct effect

Sensitivity analyses for two models with significant mediated effects revealed these findings to be moderately robust against violation of the model assumptions (data not shown).

References

1. Fox et al. SEM: Structural Equation Models. V3.1.5 R package.
2. Imai, K., Keele, L., Yamamoto, T. Identification, inference and sensitivity analysis for causal mediation effects. *Statistical Science*. 2010 (25) 51-71. DOI: 10.1214/10-STS321.
3. Tingley D., Yamamoto T., Hirose K., Keele L., Imai K. (2014). mediation: R Package for Causal Mediation Analysis. *Journal of Statistical Software*, 59(5), 1-38. URL http://www.jstatsoft.org/v59/i05/.
